# Molecular Mechanisms and Antidiabetic Effects of Mango (*Mangifera indica*) Leaf Extract as a GLP-1 Analogue in Type 2 Diabetic Rats

**DOI:** 10.3390/ijms262412149

**Published:** 2025-12-17

**Authors:** Amporn Jariyapongskul, Pornthip Boonsri, Itthipol Sungwienwong, Kulvadee Dolsophon, Nuttapon Apiratikul, Piyada Jittangprasert, Pornnapa Sitthisuk, Ruttachuk Rungsiwiwut, Siritron Samosorn, Sunit Suksamrarn, Ramida Watanapokasin

**Affiliations:** 1 Department of Physiology, Faculty of Medicine, Srinakharinwirot University, Bangkok 10110, Thailand; amporma@g.swu.ac.th; 2 Department of Chemistry and Center of Excellence for Innovation in Chemistry, Faculty of Science, Srinakharinwirot University, Bangkok 10110, Thailand; pornthipb@g.swu.ac.th (P.B.); itthipol@g.swu.ac.th (I.S.); kulvadee@g.swu.ac.th (K.D.); nuttapona@g.swu.ac.th (N.A.); piyadaj@g.swu.ac.th (P.J.); siritron@g.swu.ac.th (S.S.); sunit@g.swu.ac.th (S.S.); 3 Department of Biochemistry, Faculty of Medicine, Srinakharinwirot University, Bangkok 10110, Thailand; pornnapa.sitthisuk@g.swu.ac.th; 4 Department of Anatomy, Faculty of Medicine, Srinakharinwirot University, Bangkok 10110, Thailand; ruttachuk@g.swu.ac.th

**Keywords:** *Mangifera indica* L., glucagon-like peptide-1 (GLP-1), type 2 diabetic mellitus, hypoglycemic effect

## Abstract

This study investigated the potential of scale-up mango leaf extract (MLE) as a treatment for diabetes, a global public health concern. MLE was prepared by boiling in water, yielding 12.07% (*w*/*w*), with a bioactive mangiferin content of 165.67 ± 10.88 μg/g in the crude powder. Mechanistically, MLE demonstrated a hypoglycemic effect by stimulating glucagon-like peptide-1 (GLP-1) secretion in NCI-H716 L-cells. This occurred through activation of the MAPK signaling pathway, evidenced by increased p-ERK1/2, p-p38, and p-c-Jun expression, and the Wnt signaling pathway, shown by increased β-catenin and decreased GSK-3β and Axin1 expression, consistent with molecular docking. In a type 2 diabetic rat model, MLE administration (40 mg/kg) significantly reduced metabolic parameters, including fasting blood glucose (FBG), body weight, cholesterol (CHOL), triglycerides (TGs), and HbA1c. Notably, MLE lowered serum insulin and the HOMA-IR index, and reduced serum dipeptidyl peptidase-IV (DPP-IV) levels, resulting in increased serum GLP-1, comparable to the drug sitagliptin. These findings suggest that MLE has great potential to lower blood glucose by inducing GLP-1 secretion via MAPKs and Wnt signaling pathways, positioning it as a promising candidate for alternative diabetes treatment or development as a dietary supplement.

## 1. Introduction

Diabetes mellitus (DM) is one of the most serious chronic, noncommunicable diseases (NCDs) in the world. The prevalence of diabetes is increasing globally, with the International Diabetes Federation (IDF) estimating that around 592 million people worldwide will be affected by 2035, up from 371 million currently [1]. It also has a high mortality rate, being considered the seventh leading cause of death worldwide [2]. DM is characterized by high blood sugar (glucose) levels, known as hyperglycemia. Hyperglycemia is caused by an abnormality in either insulin function or secretion [3]. Prolonged high glucose levels can damage various parts of the body, such as the eyes and nerves, and increase the risk of kidney failure, amputations, heart attack, and stroke. Diabetes and its associated complications significantly lower people’s quality of life [4,5]. Therefore, the main goal of DM treatment is to lower blood glucose levels.

Glucagon-like peptide-1 (GLP-1) is an incretin hormone produced by enteroendocrine L-cells and secreted in response to blood glucose. It enhances insulin release in a glucose-dependent manner and suppresses glucagon secretion [6]. Currently, GLP-1-based therapies, such as GLP-1 receptor agonists (GLP-1RAs) including exendin-4 and liraglutide, are used for the treatment of type 2 diabetes (T2DM) and act similarly to GLP-1 [7]. A previous study reported that GLP-1 analogs improved hyperglycemia in patients with T2DM by stimulating the action of GLP-1 [6]. GLP-1 underlies several modern anti-diabetic therapies, highlighting the need to understand its cellular regulation. NCI-H716, a human colorectal cancer cell line that secretes GLP-1, provides a valuable in vitro model for studying L-cell (enteroendocrine) function and GLP-1 secretion [8]. The search for new anti-diabetic agents derived from natural sources, particularly plants, remains appealing because they contain bioactive compounds that may offer safer and alternative therapeutic effects against DM [9]. Medicinal plants play an important role in the prevention and treatment of DM, especially T2DM. Mango (*Mangifera indica* L.), a juicy fruit belonging to the Anacardiaceae family, is one of the most popular fruits worldwide [10]. The mango plant contains several bioactive compounds, including mangiferin, quercetin, and kaempferol o-glycoside, with mangiferin identified as the main compound responsible for anti-diabetic activity. Various parts of the mango plant—bark, leaves, roots, fruits, and flowers—have shown anti-diabetic effects. Studies have demonstrated that mango leaf extracts exhibit hypoglycemic activity in animal models [3]. In vitro, *M. indica* leaf extract (MLE) inhibited alpha-amylase activity and increased glucose uptake in LO-2 liver cells, indicating its potential to lower blood sugar levels [11]. Although the anti-diabetic effects observed in vitro and in vivo are promising, they remain to be validated in humans. Mango leaf extract has been previously investigated for its anti-diabetic properties; however, its mechanism of action on GLP-1 secretion using an in vitro L-cell model, such as NCI-H716 cells, has not yet been reported.

Therefore, this study introduces a novel approach by integrating cellular mechanistic analysis with in vivo validation and molecular docking. We aimed to investigate the effects of scale-up mango leaf extract (MLE) by traditional decoction on GLP-1 secretion and its potential to maintain hypoglycemic activity, a key feature of anti-diabetic treatment. NCI-H716, a cellular model for human enteroendocrine L cells, was used to study the cellular effects of MLE. The study also explored the molecular mechanisms underlying MLE-induced GLP-1 secretion, focusing on Wnt and MAPK signaling pathways. Additionally, the dipeptidyl peptidase-IV (DPP-IV)-inhibitory effect of MLE was evaluated in a type 2 diabetic rat model. Finally, the drug-likeness of previously reported MLE compounds and their interactions with diabetes-related enzymes were predicted using molecular docking analysis. The findings of this study may lead to the use of MLE as a functional food to regulate blood glucose levels for diabetic patients in the future.

## 2. Results

### 2.1. Mangiferin Quantification

The mangiferin content in the dried mango leaf extract powder was quantified using a developed solvent-extraction procedure coupled with high-performance liquid chromatography (HPLC). To assess extraction efficiency and reproducibility, a systematic study was conducted to optimize key solvent-extraction parameters, including solvent type (water, hot water, and ethanol), mass-to-volume ratio, and extraction time. The results revealed that the optimal conditions for extracting mangiferin from dried mango leaf consisted of a 1:10 mass-to-volume ratio of leaf powder to distilled water, with extraction performed at 100 °C for 2 h. These conditions provided the highest extraction efficiency and reproducibility. HPLC analysis showed that mangiferin exhibited a retention time of approximately 4.73 min, consistent with that of the pure mangiferin standard, confirming that the developed method is both rapid and efficient. The mangiferin content in the dried mango leaf extract powder was 165.67 ± 10.88 mg/g. This extract powder was subsequently used for further studies on molecular mechanisms as a GLP-1 hormone analogue in reducing blood glucose levels and for efficacy testing in an animal model.

### 2.2. Effect of MLE on Cell Viability in NCI-H716 Cells

**Figure 1:**
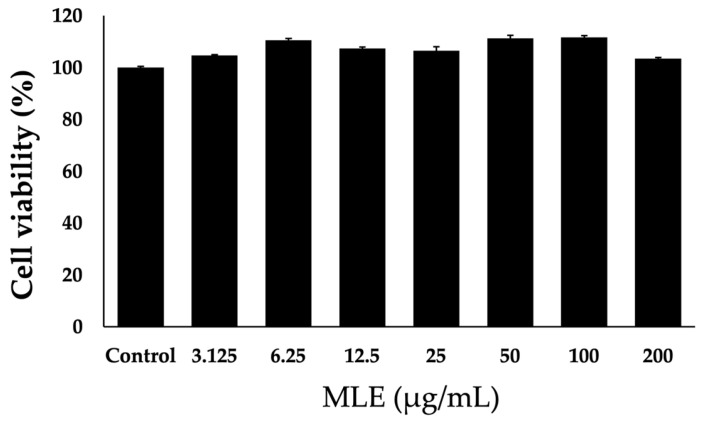
Cell viability assay of MLE on NCI-H716 cells was performed by exposing the cells to various concentrations of MLE for 24 h and assessing viability using the MTT assay. The percentage of cell viability was calculated by comparing the number of viable cells in the treated cultures with that of the untreated control cells. The data are presented as mean values ± SEM.

To evaluate the hypoglycemic effects of MLE, the initial step was assessing its impact on the cell viability of NCI-H716 cells. The cells were exposed to a range of increasing MLE concentrations, specifically from 3 to 200 µg/mL for 24 h, and subsequently analyzed using the 3-(4,5-dimethylthiazol-2-yl)-2,5-diphenyltetrazolium bromide (MTT) assay. The results demonstrated no toxicity to the NCI-H716 cells, even at the highest tested concentration, confirming that all concentrations were safe for the cells (Figure 1). Based on these findings, the concentrations of MLE selected for use in subsequent experiments were 5, 10, and 30 μg/mL.

### 2.3. MLE Triggers the Increase in GLP-1 Secretion in NCI–H716 Cells

**Figure 2:**
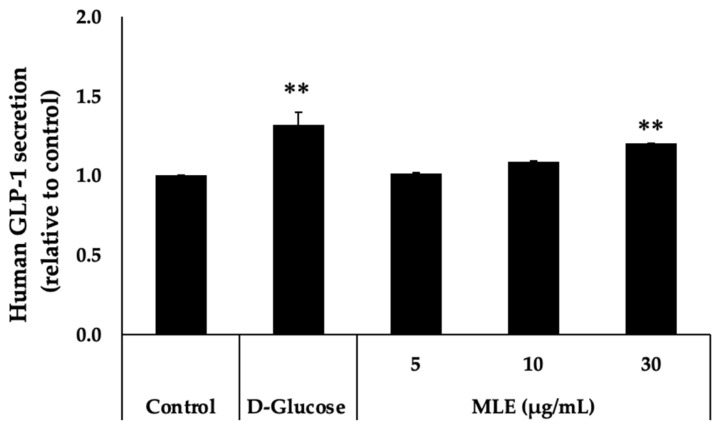
MLE-induced GLP-1 secretion from enteroendocrine NCI-H716 cells was evaluated by exposing the cells to MLE at concentrations of 5, 10, and 30 μg/mL for 2 h, followed by measurement of GLP-1 levels using a Human GLP-1 ELISA Kit (ab184857, Abcam). The results are presented relative to the control value. D-Glucose (Glc) was used as a positive control. ** *p* < 0.01 compared to the control group.

To ascertain the active role of MLE in inducing GLP-1 secretion, the cellular model of GLP-1 secretion was used. Exposure of the NCI–H716 cells for 2 h with MLE caused an increase in GLP-1 secretion at all concentrations tested (5, 10, and 30 μg/mL) in a dose-dependent manner (Figure 2).

### 2.4. Effect of MLE on Wnt/β-Catenin Signaling Pathways in NCI-H716 Cells

**Figure 3:**
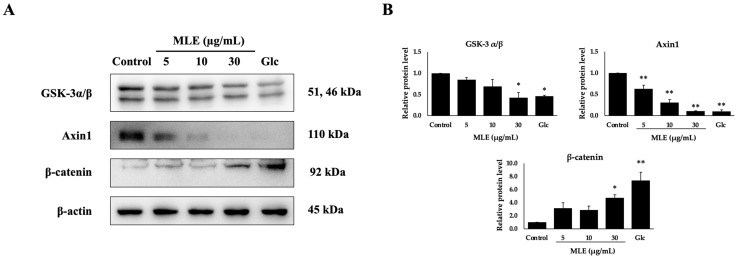
Effect of MLE on Wnt/β-catenin signaling pathway in NCI-H716 cells. Cells were treated with various concentrations of MLE (5, 10, and 30 μg/mL) and with 100 mM glucose (Glc; used as a positive control). Protein expression levels were examined by Western blot analysis (**A**). The band intensities relative to the control group are shown in (**B**). β-actin was used as the internal control. * *p* < 0.05 and ** *p* < 0.01 compared with the control group.

To begin understanding the mechanism by which MLE acts on endocrine cells, we hypothesized that its effects are mediated by the Wnt/β-catenin signaling pathway in NCI–H716 cells. We evaluated the impact of MLE at various concentrations (5, 10, and 30 μg/mL) on the protein expression of components within this pathway. As detailed in Figure 3, MLE significantly increased β-catenin expression relative to control cells. Furthermore, MLE reduced the expression levels of GSK-3α/β and Axin1, the latter of which is a potent inhibitor of β-catenin. Collectively, these findings suggest that MLE enhances GLP-1 secretion by activating the Wnt/β-catenin signaling pathway in NCI–H716 cells.

### 2.5. Effect of MLE on MAPKs Signaling Pathways in NCI-H716 Cells.

**Figure 4:**
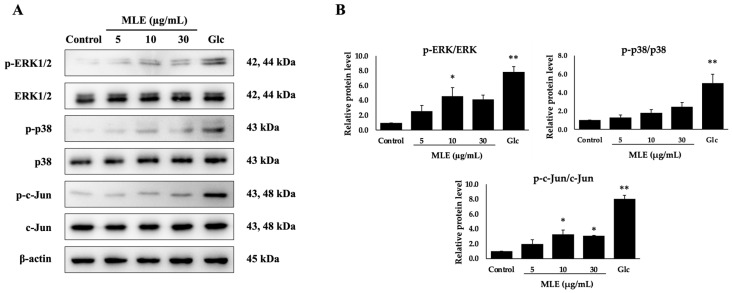
Effect of MLE on MAPK signaling pathway in NCI-H716 cells. Cells were treated with various concentrations of MLE (5, 10, and 30 μg/mL) and with 100 mM glucose (Glc; used as a positive control). Protein expression levels were examined by Western blot analysis (**A**). The band intensities relative to the control group are shown in (**B**). β-actin was used as an internal control. * *p* < 0.05 and ** *p* < 0.01 compared with the control group.

To examine the effect of MLE on intracellular pathways, we detected protein expression of ERK, p38, and c-Jun. Our results showed that MLE increased the level of p-ERK1/2, p-p38, and p-c-Jun compared to the control cells (Figure 4). This result indicates that MLE may stimulate GLP-1 secretion via activation of the MAPK pathway in NCI-H716 cells.

### 2.6. Docking Studies

**Figure A1:**
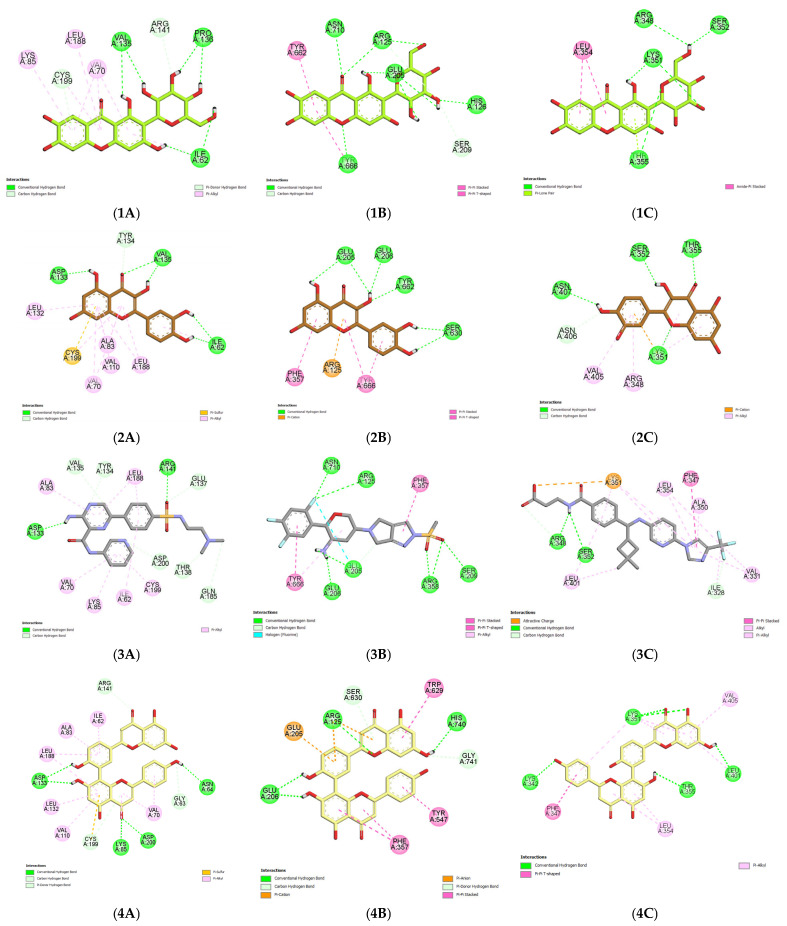

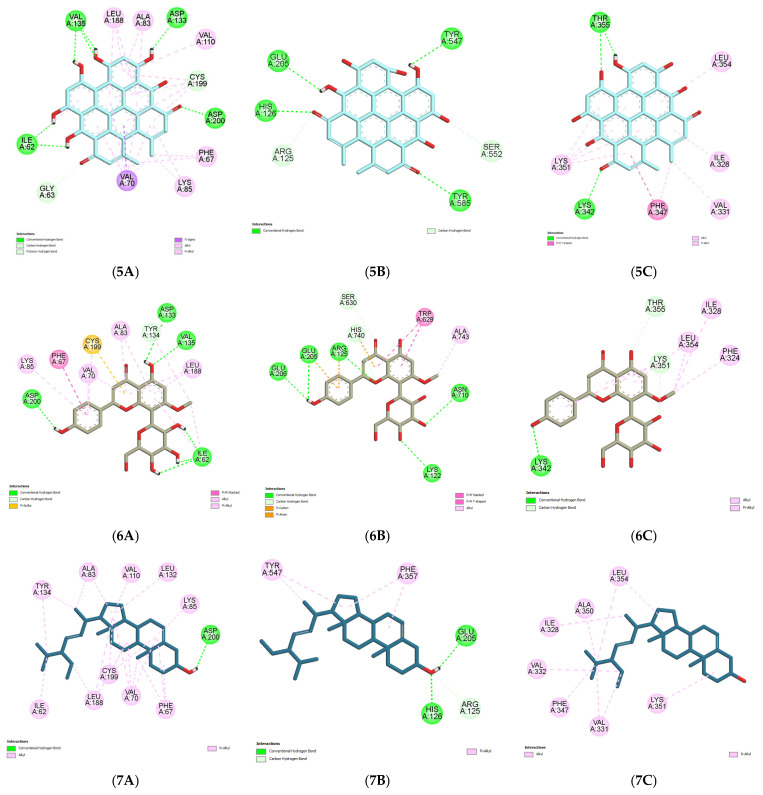
2D interacting plots between (**1**) Magiferin, (**2**) Quercetin, (**3**), Reference inhibitor, (**4**) Amentoflavone, (**5**) Hypericin, (**6**) Isoswertisin, (**7**) Stigmasterol at the binding pockets of GSK-3β (**A**), DPP-IV (**B**), and GLP-1R (**C**).

The study investigated the inhibitory potential of 27 compounds from *M. indica* leaves against three diabetes-related enzymes: GSK-3β, DPP-IV, and GLP-1R. Docking simulations confirmed that all compounds possess inhibitory activity (Table A1). As presented in Table 1, six key compounds; Mangiferin, Quercetin, Hypericin, Amentoflavone, Isoswertisin, and Stigmasterol showed binding patterns and strong binding energies comparable to the reference inhibitor, with energies ranging from 6.76 to 12.62 kcal/mol for GSK3β, 6.47 to 9.09 kcal/mol for DPP-IV, and 3.83 to −6.87 kcal/mol for GLP-1R (Table 1). The high inhibitory potential is attributed to the formation of hydrogen bonds and hydrophobic interactions with critical active site residues, which include Ile62, Val70, and Lys85 for GSK3β [12]; Glu205, Glu206, Ser209, Arg125, His126, Tyr547, Ser630, Tyr631, and His740 for DPP-IV [13]; and Lys351, Ser352, Thr355, and Asn406 for GLP-1R [14]. Importantly, Mangiferin and Quercetin, the major constituents of the leaves, exhibited binding modes and interaction profiles similar to the reference inhibitor across all three enzymes (Figure A1), suggesting they are the primary compounds responsible for the anti-diabetic potential.

### 2.7. Drug-likeness Analysis

The predicted drug-likeness properties of 27 compounds identified from *M. indica* leaves are presented in Table A2. Most compounds have a molecular weight less than 500 daltons. This variation in molecular weight can affect the absorption, distribution, metabolism, and excretion (ADME) properties, which are critical for their pharmacological efficacy. The Moriguchi octanol-water partition coefficient (MLogP) of most compounds was less than 5, indicating moderate lipophilicity, which supports membrane permeability and absorption. Generally, the number of hydrogen bond donors (<5) and hydrogen bond acceptors (<10) mostly complied with Lipinski’s guidelines. The obtained results confirmed that the active compounds from MLE showed favorable pharmacokinetic properties and were promising candidates for further drug development.

### 2.8. Effect of Mango Leaf Extract on Metabolic Parameters

**Figure 5:**
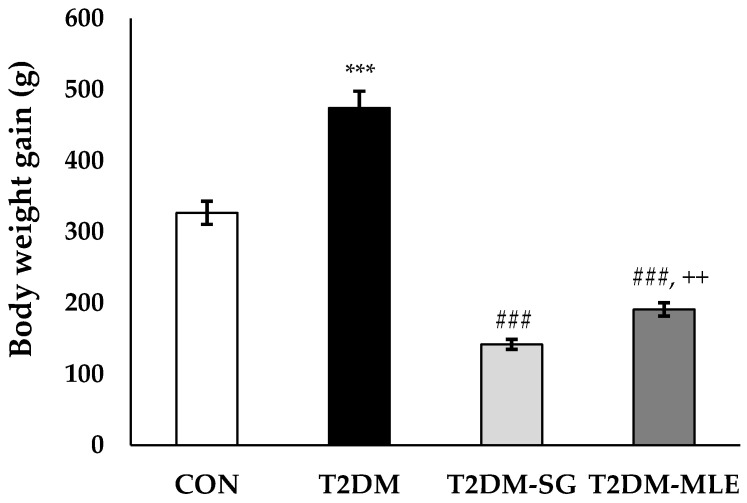
Effect of mango leaf extract (MLE) on body weight (B.W at week 12 (end of the experiment). Body weight was measured weekly throughout the 12-week experimental period in each group: CON (normal control), T2DM (type 2 diabetic rats), T2DM-SG (type 2 diabetic rats administered 10 mg/kg B.W. sitagliptin), and T2DM-MLE (type 2 diabetic rats administered 40 mg/kg B.W. mango leaf extract). Data are presented as mean ± SEM (n = 8 per group). ***, ### *p* < 0.001 vs. CON and T2DM, respectively; ++ *p* < 0.01 vs. T2DM-SG.

**Figure 6:**
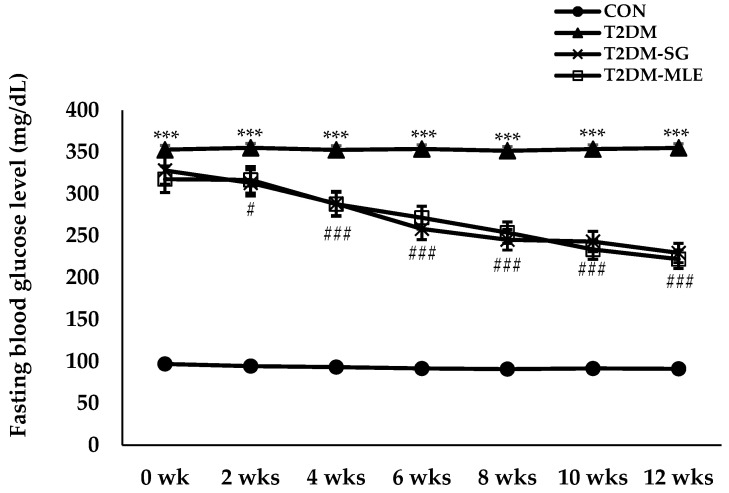
Effect of mango leaf extract (MLE) on fasting blood glucose (FBG) levels. FBG levels were measured every two weeks during the 12-week experimental period in each group: CON (normal control), T2DM (type 2 diabetic rats), T2DM-SG (type 2 diabetic rats administered 10 mg/kg B.W. sitagliptin), and T2DM-MLE (type 2 diabetic rats administered 40 mg/kg B.W. mango leaf extract). Data are presented as mean ± SEM (n = 8 per group). # *p* < 0.05 vs. T2DM; ***, ### *p* < 0.001 vs. CON and T2DM, respectively.

At the end of the 12-week experimental period, oral administration of mango leaf extract (MLE, 40 mg/kg B.W.) significantly improved all metabolic parameters in T2DM rats compared with the untreated diabetic (T2DM) group. The T2DM-MLE group markedly reduced body weight compared with the T2DM group (191.10 ± 6.22 g vs. 474.30 ± 8.59 g, *p* < 0.001, respectively), as shown in Figure 5. The fasting blood glucose (FBG) levels progressively decreased from the 4th week onward, and by week 12, FBG levels were significantly lower in the T2DM-MLE group compared with the T2DM group (220.20 ± 8.39 vs. 355.00 ± 13.41 mg/dL, *p* < 0.001, respectively), as shown in Figure 6. In addition, the T2DM-MLE group significantly reduced glycosylated hemoglobin (HbA1c), total cholesterol (CHOL), and triglyceride (TG) levels compared with the T2DM group (HbA1c: 7.72 ± 0.18% vs. 12.26 ± 0.55%; CHOL: 162.40 ± 1.17 vs. 185.20 ± 2.06 mg/dL; TG: 380.80 ± 13.82 vs. 512.40 ± 17.83 mg/dL, *p* < 0.001, respectively), as shown in Table 2. Although the HbA1c and TG in the T2DM-MLE group were higher than those observed in the sitagliptin-treated (T2DM-SG) group, it still showed a marked and statistically significant reduction compared with the T2DM group.

### 2.9. Effect of Mango Leaf Extract on Glucose Tolerance

**Figure 7:**
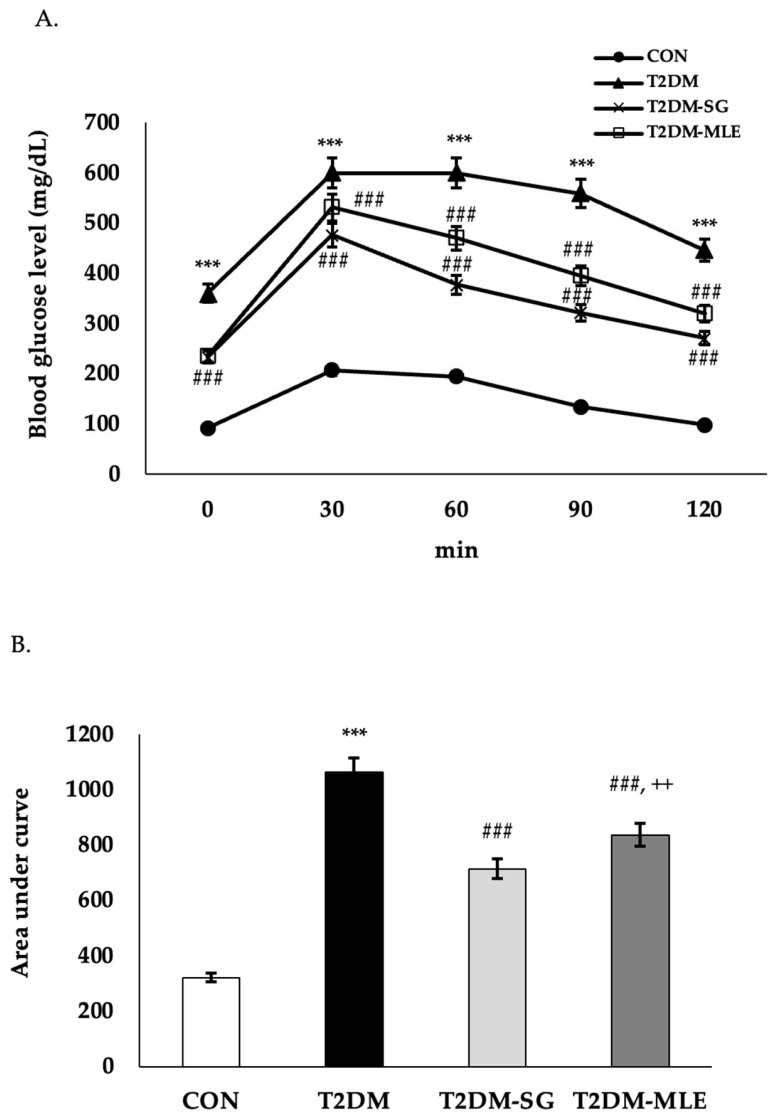
Effect of mango leaf extract (MLE) on blood glucose levels during the oral glucose tolerance test (OGTT; (**A**)) and the area under the curve (AUC; (**B**)) of OGTT in each group: CON (normal control), T2DM (type 2 diabetic rats), T2DM-SG (type 2 diabetic rats administered 10 mg/kg B.W. sitagliptin), and T2DM-MLE (type 2 diabetic rats administered 40 mg/kg B.W. mango leaf extract). The OGTT was performed at week 12 after MLE and SG administration. Blood glucose levels were measured at 0 (baseline), 30, 60, 90, and 120 min after glucose loading. Data are presented as mean ± SEM (n = 8 per group). ***, ### *p* < 0.001 vs. CON and T2DM, respectively; ++ *p* < 0.01 vs. T2DM-SG.

During the oral glucose tolerance test (OGTT), the T2DM group exhibited persistently elevated blood glucose levels throughout the 120-min test period. In contrast, the T2DM-MLE group showed a significant reduction in postprandial glucose levels at all time points compared with the T2DM group (*p* < 0.001), as shown in Figure 7A. When comparing the area under the glucose curve (AUC) among treatment groups (Figure 7B), the T2DM-MLE group exhibited a significantly lower AUC than the untreated diabetic (T2DM) group, indicating improved glucose tolerance. The AUC value in the T2DM-MLE group was higher than that observed in the sitagliptin-treated (T2DM-SG) group. However, it still showed a marked and statistically significant reduction compared with the T2DM group.

### 2.10. Effect of Mango Leaf Extract on Serum Insulin and Insulin Resistance

**Figure 8:**
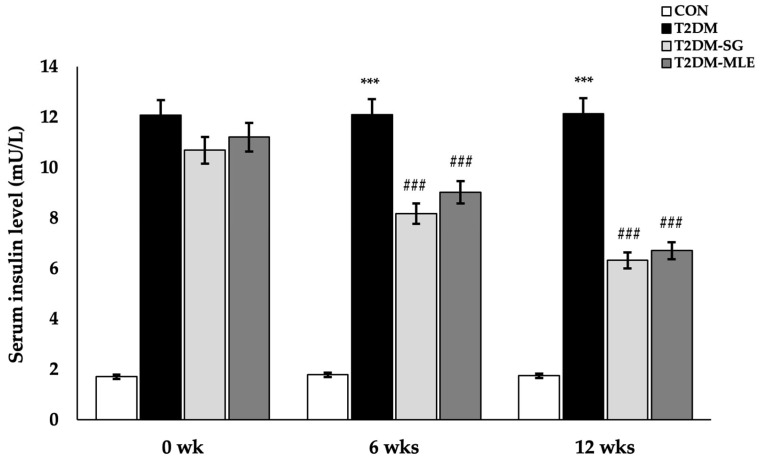
Effect of mango leaf extract (MLE) on serum insulin levels. Serum insulin levels were determined at baseline (week 0), week 6, and week 12 of the experimental period in each group: CON (normal control), T2DM (type 2 diabetic rats), T2DM-SG (type 2 diabetic rats administered 10 mg/kg B.W. sitagliptin), and T2DM-MLE (type 2 diabetic rats administered 40 mg/kg B.W. mango leaf extract). Data are presented as mean ± SEM (n = 8 per group). ***, ### *p* < 0.001 vs. CON and T2DM, respectively.

To assess insulin resistance, serum insulin levels were measured, and the homeostatic model assessment for insulin resistance (HOMA-IR) was calculated using serum insulin and fasting blood glucose (FBG) values as described in the methods section. Figure 8 shows serum insulin levels at 0, 6, and 12 weeks after MLE administration. MLE administration in the T2DM-MLE group for six weeks significantly reduced serum insulin levels compared with the T2DM group (9.02 ± 0.24 vs. 12.10 ± 0.41 mU/L, *p* < 0.001), and this reduction persisted through week 12 (6.70 ± 0.35 mU/L). After 12 weeks, the HOMA-IR index in the T2DM-MLE group was significantly lower than that in the untreated T2DM group (3.71 ± 0.33 vs. 10.68 ± 0.75, *p* < 0.001, respectively) (Table 3). Furthermore, serum insulin and HOMA-IR values in the T2DM-MLE group did not differ significantly from those in the T2DM-SG group. These findings indicate that MLE exerts insulin-sensitizing effects comparable to those of sitagliptin.

### 2.11. Effect of Mango Leaf Extract on Serum Dipeptidyl Peptidase-IV (DPP-IV) and Glucagon-like Peptide-1 (GLP-1)

**Figure 9:**
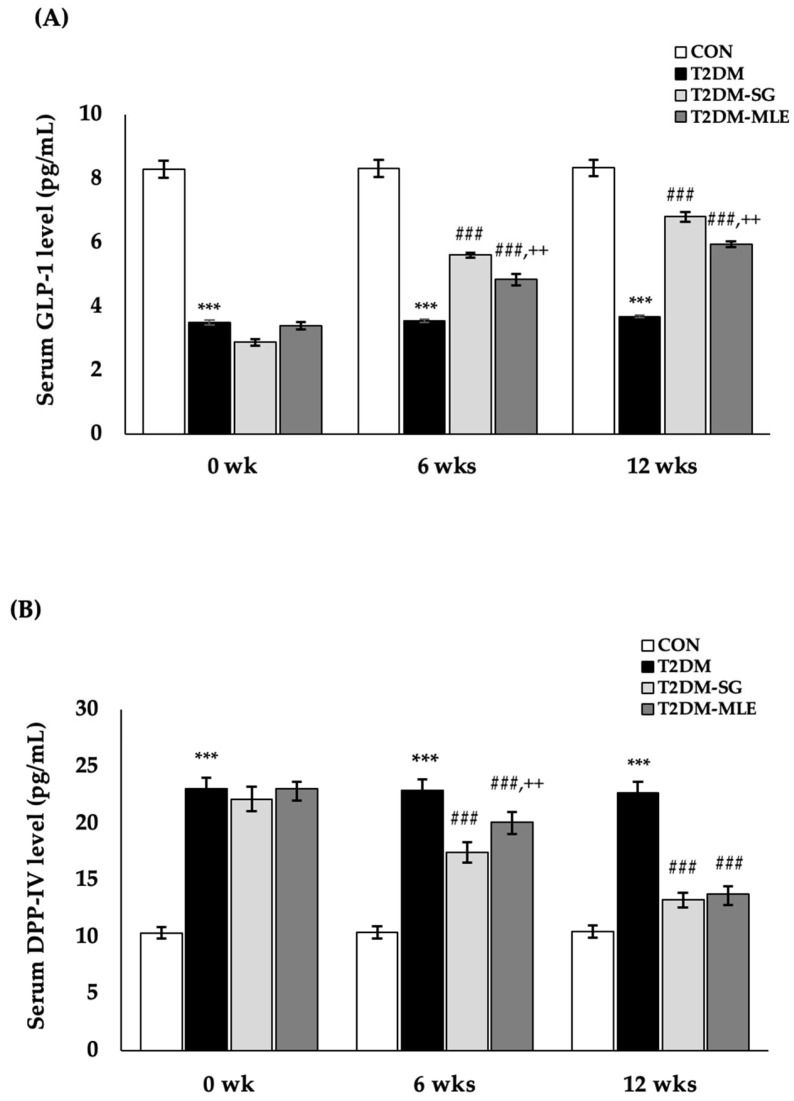
Effects of mango leaf extract (MLE) on serum glucagon-like peptide-1 (GLP-1; (**A**)) and dipeptidyl peptidase-4 (DPP-IV); (**B**)) levels. Serum GLP-1 and DPP-IV levels were measured at baseline (week 0), week 6, and week 12 of the experimental period in each group: CON (normal control), T2DM (type 2 diabetic rats), T2DM-SG (type 2 diabetic rats administered 10 mg/kg B.W. sitagliptin), and T2DM-MLE (type 2 diabetic rats administered 40 mg/kg B.W. mango leaf extract). Data are presented as mean ± SEM (n = 8 per group). ***, ### *p* < 0.001 vs. CON and T2DM, respectively; ++ *p* < 0.01 vs. T2DM-SG.

To evaluate the effect of MLE on incretin-related biomarkers, serum dipeptidyl peptidase-IV (DPP-IV) and glucagon-like peptide-1 (GLP-1) levels were measured at 0, 6, and 12 weeks after MLE administration. As shown in Figure 9A, the T2DM-MLE group exhibited significantly higher serum GLP-1 levels compared with the untreated T2DM group at both time points (6 weeks: 4.85 ± 0.18 vs. 3.55 ± 0.05 pg/mL; 12 weeks: 5.95 ± 0.09 vs. 3.69 ± 0.04 pg/mL; *p* < 0.001, respectively). As shown in Figure 9B, serum DPP-IV levels in the T2DM-MLE group were significantly decreased at both 6 and 12 weeks compared with the T2DM group (6 weeks: 20.09 ± 0.94 vs. 22.89 ± 0.56 pg/mL; 12 weeks: 13.80 ± 0.64 vs. 22.67 ± 0.49 pg/mL; *p* < 0.001, respectively). Furthermore, although serum GLP-1 and DPP-IV levels in the T2DM-MLE group did not reach the levels observed in the sitagliptin-treated (T2DM-SG) group, MLE treatment still produced significant improvements in incretin-related parameters. These findings indicate that MLE exerts notable incretin-modulating effects and possesses potential DPP-IV inhibitory activity that enhances circulating GLP-1 levels in T2DM rats.

## 3. Discussion

Medicinal plants have increasingly been reported for their potential in preventing and/or treating diabetes worldwide through various mechanisms, including lowering blood glucose levels. The hypoglycemic effects of several plants traditionally used as antidiabetic remedies have been validated, and their underlying mechanisms continue to be investigated [9]. In traditional medicine, the dried leaves of the mango tree (*Mangifera indica* L.) have long been recognized for their usefulness in managing diabetes and respiratory infections [15]. Most medicinal plants contain a range of phytochemicals, such as polyphenols (including flavonoids and anthocyanins), carotenoids, and others, that are frequently associated with antidiabetic effects [16]. Several studies have reported that mango leaves contain multiple bioactive compounds, including mangiferin, quercetin, and kaempferol *O*-glycosides [3]. This study specifically examines the antidiabetic potential of natural compounds in MLE. Our findings demonstrate that MLE stimulates GLP-1 secretion in enteroendocrine NCI-H716 cells, consistent with the well-established role of GLP-1 in lowering blood glucose levels and exerting antihyperglycemic effects. Recent reports have shown that methanolic extracts of mango leaves inhibit DPP-IV activity and enhance GLP-1 secretion in vitro in models of type 2 diabetes mellitus (T2DM) [17]. Furthermore, the combination of the DPP-IV inhibitor sitagliptin and mangiferin—one of the major phytochemicals in mango—significantly improved glucose tolerance and increased plasma insulin and active GLP-1 levels in streptozotocin-induced diabetic rats [18].

GLP-1, an incretin hormone released from intestinal L cells, plays a central role in maintaining glucose homeostasis through multiple molecular pathways. It exerts antihyperglycemic effects by stimulating glucose-dependent insulin secretion, suppressing glucagon release, reducing hepatic glucose production, and decreasing appetite [19]. Recent evidence suggests that GLP-1 secretion can be modulated by effectors of the Wnt signaling pathway [20]. The canonical, β-catenin-dependent Wnt pathway regulates key cellular processes—including proliferation, survival, migration, and differentiation. Activation begins when Wnt ligands bind to Frizzled receptors and the coreceptors LRP5/6, leading to activation of the intracellular protein Dishevelled (Dvl). Dvl inhibits the β-catenin destruction complex, composed of Axin, adenomatosis polyposis coli (APC), protein phosphatase 2A (PP2A), glycogen synthase kinase 3 (GSK3), and casein kinase 1α (CK1α). This inhibition allows β-catenin to accumulate and translocate into the nucleus, where it binds members of the LEF/TCF transcription factor family, including TCF7L2, to activate Wnt target gene expression [21].

GLP-1 is encoded by the proglucagon gene (gcg). Previous studies have shown that two major Wnt pathway effectors—β-catenin and TCF7L2—specifically upregulate gcg mRNA expression and GLP-1 production in intestinal L cells [22]. In this study, we evaluated the influence of MLE on the Wnt signaling pathway. Our results show that MLE increased β-catenin expression while reducing levels of GSK3β and Axin1, both of which are negative regulators of β-catenin. These findings are consistent with Kim M-H et al. (2014), who reported that metformin—a widely used T2DM medication—enhances GLP-1 secretion by promoting β-catenin nuclear translocation and transcription of the Wnt-responsive gene glu (a GLP-1 precursor), mediated through activation of the insulin signaling pathway and subsequent inhibition of GSK3β in NCI-H716 cells and db/db mice [23].

Mitogen-activated protein kinase (MAPK) signaling pathways regulate numerous cellular processes, including proliferation, differentiation, apoptosis, and stress responses. The major MAPK pathways include extracellular signal-regulated kinase 1/2 (ERK1/2), c-Jun N-terminal kinase (JNK), and p38 MAPK [24]. Activation of intracellular MAPK pathways has been proposed as a requirement for GLP-1 secretion [25]. In this study, MLE increased levels of phosphorylated ERK1/2 (p-ERK1/2), phosphorylated p38 (p-p38), and phosphorylated c-Jun (p-c-Jun). Many studies support the involvement of MAPK signaling in GLP-1 secretion. Berberine, the primary active compound in Rhizoma coptidis, reduces blood glucose and insulin levels in diabetic rats via MAPK and GnRH-GLP-1 pathways in the ileum [26]. Ezetimibe, a cholesterol-lowering drug, has been shown to stimulate active GLP-1 secretion through MEK/ERK activation and to suppress GLP-1 secretion when MEK is inhibited by PD98059 [27]. Reimer RA (2006) reported that meat hydrolysates enhance GLP-1 secretion via ERK1/2 activation in NCI-H716 cells, while essential amino acids increase GLP-1 secretion through activation of ERK1/2 and p38 [28]. Short-chain fatty acids (SCFAs)—including acetate, propionate, and butyrate—have also been reported to increase GLP-1 expression via p-ERK and p-p38 activation, with butyrate additionally upregulating p-JNK [29]. However, future studies employing selective inhibitors or gene-silencing approaches are necessary to determine whether these pathways are essential for MLE-induced GLP-1 secretion.

Based on the in vitro findings, an in vivo study was conducted to confirm the biological effects of MLE in a T2DM rat model. Following a previously validated protocol [30], rats were administered 10% (*w*/*v*) fructose in their drinking water and then received a single intravenous injection of STZ (30 mg/kg B.W.). Successful induction of T2DM was verified by abnormal metabolic parameters and elevated serum insulin and HOMA-IR values, as summarized in Table 2 and Table 3 and Figure 5, Figure 6, Figure 7 and Figure 8.

In this study, treatment with MLE (40 mg/kg B.W.) for 12 weeks significantly reduced fasting blood glucose (FBG), HbA1c, total cholesterol, triglycerides, serum insulin, and HOMA-IR values in T2DM rats compared with untreated diabetic controls. These results indicate that MLE improves glycemic control and lipid metabolism while enhancing insulin sensitivity. The magnitude of improvement was comparable to that observed with sitagliptin (10 mg/kg B.W.), a standard DPP-IV inhibitor used as a positive control. The antihyperglycemic effects of MLE are consistent with previous findings demonstrating that *M. indica* leaf extracts reduce blood glucose and lipid levels in diabetic animal models [31,32]. Ojewole JAO (2005) and S. Muruganandan et al. (2005) similarly reported that methanolic and ethanolic extracts of mango leaves lower FBG, triglycerides, and total cholesterol while improving insulin sensitivity in STZ-induced diabetic rats [33,34].

In the present study, MLE also markedly improved oral glucose tolerance, as reflected by reduced postprandial glucose levels and area under the curve (AUC) during the OGTT, indicating improved glucose utilization and peripheral insulin sensitivity. Improvements in insulin resistance were further supported by reductions in serum insulin and HOMA-IR after 6 and 12 weeks of treatment. These findings align with previous reports that *Mangifera indica* leaf extract enhances insulin sensitivity by promoting GLUT4 translocation and activating AMP-activated protein kinase (AMPK) signaling, thereby improving glucose uptake and energy metabolism [35,36].

Notably, MLE treatment significantly increased circulating GLP-1 levels while reducing DPP-IV activity, indicating its potential dual role as both a DPP-IV inhibitor and a GLP-1 enhancer. Elevated DPP-IV activity is known to contribute to insulin resistance and hyperglycemia in T2DM [37,38]. DPP-IV inhibitors such as sitagliptin improve glycemic control by prolonging the half-life of active GLP-1 and enhancing glucose-dependent insulin secretion [39]. The comparable effects of MLE and sitagliptin observed in this study suggest that the antidiabetic effects of MLE may involve increasing circulating GLP-1 levels through inhibition of DPP-IV.

Molecular docking prediction studied results showed that all 27 active compound molecules from mango leaves could bind to the key interacting residues of 3 diabetes-causing enzymes, including GSK3β, DPP-4, and GLP-1R with the promising binding affinities (Table A1). Interestingly, the major compounds like mangiferin and quercetin exhibited the similar binding modes to GSK3β, DPP-IV and GLP-1R comparing to native ligand-enzymes complexes (Figure A1). These findings are consistent with in vivo results, confirming that the active compounds from MLE can inhibit DPP-IV and GSK3β, as well as enhance GLP-1 secretion. Furthermore, most of the predicted drug-likeness properties, 11β-HSD1 and GSK-3β, aligned with Lipinski’s rule of five, with the exceptions of Caffeic acid, Shikimic acid, Theogallin, Kaempferol 3-*O*-rutinoside, Catechin and Taxifolin, which exhibit good drug-likeness properties. However, since these compounds are present in low quantities within the active constituents of mango leaves, they are unlikely to have a significant effect on antidiabetic activity as compared to mangiferin and quercetin. Finally, the computational prediction results suggest that the active compounds from MLE may serve as potent inhibitors for the treatment of type 2 diabetes mellitus (T2DM).

Taken together, our results demonstrate for the first time the ability of mango leaf extract to stimulate GLP-1 secretion in NCI-H716 L-cells, a mechanism not previously reported in the literature. Our combined in vitro, in vivo, and in silico findings provide new mechanistic insights into how mango leaf extract may exert antidiabetic effects through the enteroendocrine L-cell pathway.

## 4. Materials and Methods

### 4.1. Reagents and Materials

Streptozotocin (STZ) was obtained from Merck (Billerica, MO, USA). Glucose, cholesterol, and triglyceride strips were purchased from Roche (Mannheim, Germany). The insulin assay kit was purchased from Merck (Billerica, MO, USA). The DPP-IV and GLP-1 assay kits were purchased from CUSABIO (Houston, TX, USA). The fructose powder was purchased from P.S. Foods and Chemical (Bangkok, Thailand).

### 4.2. Plant Material and Extraction

Fresh organic mango leaves were collected from Klong Takrao, Tha Takiap, Chachoengsao, Thailand. The leaves were cleaned and dried in a hot air oven at 60–70 °C, and then ground into a fine powder and passed through no. 80 mesh sieve. The dried mango leaf powder was mixed with distilled water in a 1:10 mass-to-volume ratio. This mixture was subsequently heated at 100 °C for 2 h. The mixture was filtered using Whatman no. 91 filter paper to separate the solid residue. The resulting aqueous filtrate was then dried by a spray dryer to give the crude mango leaf extract (MLE) powder (12.07% *w*/*w*). For the large-scale extraction, a standardized ratio of dried leaf powder to distilled water (1:10 *w*/*v*) was maintained for all batches. The mixture was heated to 100 °C and maintained under constant stirring for 2h. This hot-water extraction process was performed on a 300 kg batch scale. The obtained MLE was kept in a vacuum-sealed aluminum foil bag at 3–5 °C for further investigations.

### 4.3. HPLC Analysis

Prior to HPLC analysis, the MLE powder was dissolved in deionized water. The suspension was subsequently centrifuged and filtered through Whatman No. 1 filter paper to obtain a clear supernatant. The filtered samples were then diluted to an appropriate concentration and passed through a 0.22 µm syringe filter before injection. Mangiferin content in MLE powder was analyzed by our developed HPLC method using an LC-20 Prominence Series system (Shimadzu, Kyoto, Japan). The separation of mangiferin was achieved using a C18 column (Inertsil ODS-3, 150 × 4.6 mm, 5.0 µm). The mobile phase consisted of 0.10% acetic acid and ethanol (75:25 *v*/*v*) at a flow rate of 1.00 mL/min. The analyte was detected at a wavelength of 316 nm using a diode array detector (DAD). Mangiferin concentrations were determined from a standard curve constructed using five concentrations of a mangiferin standard (purity > 98%). The mangiferin content was expressed in units of milligrams of mangiferin per gram of dried mango leaf extracted powder (mg/g).

### 4.4. Cell Culture

Human NCI-H716 cell line was obtained from the American Type Culture Collection (ATCC, Manassas, VA, USA). The cells were cultured in suspension using Roswell Park Memorial Institute (RPMI) 1640 Medium with high glucose, supplemented with penicillin (100 U/mL), streptomycin (100 µg/mL), and 10% fetal bovine serum (FBS). They were maintained in a CO_2_ incubator at 37 °C with 5% carbon dioxide and 95% humidity. The culture medium was replaced every 5 to 7 days.

### 4.5. Cell Cytotoxicity by MTT Assay

NCI-H716 cells were seeded on a 96-well plate for 24 h. The cells were treated with different concentrations of MLE for 24 h, whereas DMSO was used as the control group. At the end of 24 h incubation, the medium in each well was replaced with MTT solution (0.5 mg/mL), and the plates were incubated for 2–4 h. MTT solution was removed, and the formazan crystals produced by viable cells were dissolved in DMSO. The absorbance was then determined at 570 nm by using a microplate reader (Multiskan Sky Microplate Spectrophotometer, Thermo Fisher Scientific, Waltham, MA, USA). The non-toxic concentration was chosen for further experiments.

### 4.6. GLP-1 Secretion Studies

NCI-H716 cells were seeded on a 24-well plate coated with Matrigel and grown until confluence. Then, the supernatant was replaced by Krebs-Ringer bicarbonate buffer with or without MLE and incubated for 2 h at 37 °C in a humidified incubator at 5% CO_2_, whereas 100 mM D-glucose was used as the positive control. The supernatant was then collected and centrifuged at 4 °C for 10 min to remove cell debris. The GLP-1 secretion in the supernatants was determined using Human Simple Step ELISA^®^ Kit (ab184857, Abcam, Waltham, MA, USA).

### 4.7. Western Blot Analysis

Protein expression was detected by Western blot analysis. Proteins were separated using SDS-polyacrylamide gel electrophoresis (PAGE) and then transferred onto polyvinylidene difluoride (PVDF) membranes employing a Mini Trans-Blot Cell^®^ (Bio-Rad Laboratories, Hercules, CA, USA). The membrane was blocked by 5% BSA and probed with primary antibody targeting specific proteins (Cell Signaling Technology, Beverly, MA, USA.) at 4 °C, respectively. After washing three times with TBST, the membrane was incubated with secondary antibody conjugated with horseradish peroxidase (Cell Signaling Technology, Beverly, MA, USA.) for 1 h at room temperature. The signals were detected by using Immobilon™ Western Chemiluminescent HRP Substrate (ECL) (Merck Millipore Corp., Darmstadt, Germany) and quantified by densitometry with ImageJ software version 1.53e.

### 4.8. Animals

Male Spraque-Dawley (SD) rats (N = 32, age 6 weeks, weighted 150–189 g) were obtained from Nomura Siam International (Bangkok, Thailand). The rats were maintained in plastic cages (N = 2–3 rats/cage) under a temperature of 20 ± 2 °C, 60% humidity, and 12 h of light/dark cycle. A week after, rats were randomly divided into four groups (N = 8), including the normal control (CON) rats, type 2 diabetic (T2DM) rats, type 2 diabetic rats administered with 10 mg/kg B.W. of sitagliptin (T2DM-SG; positive control), and type 2 diabetic rats administrated with 40 mg/kg B.W. of mango leaf extract (T2DM-MLE) groups, respectively. The MLE 40 mg/kg B.W. dose was selected based on previous in vivo studies, which demonstrated antihyperglycemic and organ-protective effects of mangiferin (the major bioactive constituent of mango leaf) [36,40]. All of the experiments were performed in compliance with the regulations approved by the ethics committee of Srinakharinwirot University (COA/AE-003-2022).

### 4.9. Type 2 Diabetic Model Induction

The type 2 diabetic rat model was induced by 10% fructose drinking water feeding combined with 30 mg/kg body weight of streptozotocin (STZ) intravenous (I.V.) injection as the method described by Wilson, R. D and Islam M [30]. A week after acclimatization, the rats received 10% fructose drinking water for two weeks following 30 mg/kg B.W. of STZ injection. After 48 h of STZ injection, the fasting blood glucose (FBG) was monitored. The rats with FBG levels ≥ 250 mg/dL were considered as diabetes. After the rats were considered as type 2 diabetes, sitagliptin (SG) and mango leaf extract (MLE) were administered for 12 weeks. SG and MLE were prepared in distilled water (DW). CON rats were ad libitum fed with normal drinking water, while all T2DM groups were fed with 10% fructose drinking water throughout the experiment.

### 4.10. Metabolic Parameters Detection

At the end of the experiment, the metabolic parameters including body weight (B.W.), fasting blood glucose (FBG), blood cholesterol (CHOL), blood triglyceride (TG), plasma HbA1c, and serum insulin were evaluated in all groups. FBG, CHOL, and TG were measured using a glucometer and cholesterol/triglyceride strip Accu-trend^®^ GCT meter (Roche, Germany), respectively. Plasma HbA1c was determined by turbidimetric immune inhibition assay (Professional Laboratory Management Crop. Co., Ltd., Bangkok, Thailand).

### 4.11. Effect of Mango Leaf Extract on Serum Insulin and Insulin Resistance

Serum insulin was evaluated by an insulin assay kit (Merck, Billerica, MO, USA) under the manufacturer’s instructions, respectively. In addition, the homeostasis model assessment of insulin resistance (HOMA-IR) index is used to evaluate insulin sensitivity, which was carried out by the following formula: HOMA-IR = (FBG × Insulin)/22.5 [41].

### 4.12. Oral Glucose Tolerance Test

In the 11th week of the experimental period, an oral glucose tolerance test (OGTT) was performed in all groups by administering a single gavage dose of a 50% glucose solution (2 g/kg B.W.). Blood samples were collected before glucose administration (baseline = 0 min) and at 30, 60, 90, and 120 min afterward. Blood glucose levels were measured using a glucometer. The area under the curve (AUC) of the OGTT was calculated to represent overall glucose tolerance.

### 4.13. Serum Glucagon-like Peptide 1 and Dipeptidyl Peptidase-IV Detection

At the end of the experiment, the serum glucagon-like peptide 1 (GLP-1) and dipeptidyl peptidase-IV (DPP-IV) enzyme were measured by an assay kit (CUSABIO, USA).

### 4.14. Statistical Analysis

The data in molecular mechanism studies were presented as the mean ± standard error of the mean. Differences between groups were determined using the One-way analysis of variance (ANOVA). Statistical significance is defined as *p*-values < 0.05 by using SPSS statistical software package (version 20.0). Similarly, the data presented in animal model were analyzed using Graph Pad Prism software (version 7.0) and presented as the mean ± standard error of the mean (Mean ± SEM). The differences in the mean of metabolic parameters, OGTT area under the curve, serum insulin, HOMA-IR, serum GLP-1, and serum DPP-IV were analysed by one-way ANOVA, followed by Tukey’s post-hoc test. Statistical significance was defined as a *p*-value ≤ 0.05.

### 4.15. Ligand Identification and Optimization

*Phytocompounds of M. indica* leaves were identified from a study of Kumar, M. et al. in 2021 [42]. Twenty-seven compounds belonging to xanthone, flavonoids, and phenolics were selected for study, and their structures were retrieved from the PubChem database (https://pubchem.ncbi.nlm.nih.gov/ (accessed on 5 February 2022)). Then, the structures were optimized using Gaussian 09 program [43].

### 4.16. Molecular Docking

The 3D structure of the GSK3β, DPP-IV, and GLP-1R with PDB IDs 4ACC, 4PNZ, and 5VEW, respectively, was obtained from the protein databank (https://www.rcsb.org/ (accessed on 18 February 2022)) [13,14,44,45,46,47,48]. The molecular docking analyses of the 27 selected compounds were screened using AutoDock Tools (ADT 1.5.6) and AutoDock 4.2, available from the Scripps Research Institute (http://www.scripps.edu/mb/olson/doc/autodock (accessed on 8 October 2019)) [45]. AutoDock was run using a grid box created with 60 × 40 × 40 points centered on the ligand. Then, Gasteiger-type polar hydrogen charges were assigned, and the torsions were set on the selected molecules. It was followed by assigning the Gasteiger charges and the addition of atomic solvation parameters. The 27 compounds were docked into each enzyme. Binding energy for different complexes obtained was used for the initial evaluation of the result. A cluster analysis, the pose with the lowest score was selected from conformations, which was selected as the most reliable solution. The ligand protein interactions were created by using the Discovery Studio version 2021 (BIOVIA, San Diego, CA, USA) [46].

### 4.17. Drug-likeness Prediction

The drug-like properties of 27 compounds identified from MLE were predicted using the SwissADME web tool [47].

## 5. Conclusions

In summary, the results of the present study suggest that MLE stimulates GLP-1 secretion from NCI-H716 cells. The effects of MLE on the GLP-1 secretion are dependent on the MAPKs and Wnt/β-catenin signaling pathway. Moreover, the long-term oral administration of MLE had a potential treatment effect on hyperglycemia, hyperinsulinemia, and metabolic profile alteration in the T2DM rat model by inhibiting DPP-IV and activating GLP-1 secretion. Therefore, MLE may be potentially effective for the prevention and treatment of diabetes. However, further studies, particularly clinical trials, are required to confirm its efficacy and safety before MLE can be considered for practical applications in diabetes management.

## Figures and Tables

**Table 1:** Binding energy, BE (kcal/mol) and the interacting residues of the selected compounds against the identified targets of diabetes. Key interacting residues are indicated in bold to emphasize their critical role in ligand–target binding.

Compound	GSK3β			DPP-IV			GLP-1R		
	BE	Hydrogen Bond	Pi-Alkyl	BE	Hydrogen Bond	Pi-Alkyl/Pi-Pi Stacked	BE	Hydrogen Bond	Pi-Alkyl/Pi-Pi
Hypericin	−12.62	**Ile62**, Asp133, Val135, Asp200	**Val70**, Ala83, **Lys85**, Val110, Leu188, Cys199	−7.45	His126, **Glu205**, **Tyr547**, Cys551, Tyr666		−4.94	Lys342, **Thr355**	Ile328, Val331, **Lys351**, Leu354/Phe347
Amentoflavone	−10.15	Asn64, **Lys85**, Asp133	**Val70**, Ala83, **Lys85**, Val110, Leu132, Leu188	−9.09	**Glu206, Arg125, His740**	/Phe357, **Tyr547**, Trp629	−6.68	Lys342, **Lys351**, **Thr355**, Leu401	**Lys351**, Leu354, Leu401, Val405/Phe347
Isoswertisin	−9.30	**Ile62**, Asp133, Val135, Asp200	**Ile62**, **Val70**, Ala83, Lys85, Leu188, Cys199	−7.88	Lys122, **Arg125**, **Glu205**, **Glu206**, Asn710	Ala743/Trp629	−4.91	Lys342	Ile328, Phe324, **Lys351**, Leu354
Stigmasterol	−10.22	Asp200	Phe67, **Ile62**, **Val70**, **Lys85**, Ala83, Val110, Leu132 Leu188,	−9.05	**His126, Glu205**	**His126, Glu205**	−6.87		Ile328, Val331, Val332, Phe347, Ala350, **Lys351**, Leu354
Mangiferin	−6.76	Val135, Pro136, **Ile62**	**Val70**, **Lys85**, Leu188	−6.47	**Arg125**, **His126**, **Glu205**, Tyr666, Asn710	/Tyr662, Tyr666	−3.83	Arg348, **Lys351**, **Ser352**, **Thr355**	Leu354
Quercetin	−7.06	**Ile62**, Asp133, Val135	**Val70**, Ala83, Val110, Leu132, Leu188	−6.62	**Glu205**, **Glu206**, **Ser630**, Tyr662	/Phe357, Tyr666	−5.28	**Lys351**, **Ser352**, **Thr355**, Asn407	Arg348, **Lys351**, Val405
Reference inhibitor	−9.28	Asp133, Arg141	**Ile62**, **Val70**, Ala83, **Lys85**, Leu188, Cys199	−10.94	**Arg125**, **Glu205**, **Glu206**, **Ser209**, Arg358, Asn710	Tyr666/Tyr666, Phe357	−6.28	Arg348, **Ser352**	Val331, Ala350, **Lys351**, Leu354, Leu401/Phe347

**Table 2:** Plasma glycosylated hemoglobin (HbA1c), total blood cholesterol (CHOL), and blood triglyceride (TG) levels at week 12 (end of the experiment) in each group: CON (normal control), T2DM (type 2 diabetic rats), T2DM-SG (type 2 diabetic rats administered 10 mg/kg B.W. sitagliptin), and T2DM-MLE (type 2 diabetic rats administered 40 mg/kg B.W. mango leaf extract).

	HbA1c (%)	CHOL (mg/dL)	TG (mg/dL)
CON	3.84 ± 0.05	150.60 ± 0.40	110.80 ± 1.02
T2DM	12.26 ± 0.55 ***	185.20 ± 2.06 ***	512.40 ± 17.83 ***
T2DM-SG	5.42 ± 0.43 ^###^	158.40 ± 0.75 ^###^	301.60 ± 10.23 ^###^
T2DM-MLE	7.72 ± 0.18 ^###, ++^	162.40 ± 1.17 ^###^	380.80 ± 13.82 ^###, ++^

The data are presented as mean ± SEM (n = 8 per group). ***, ^###^ *p* < 0.001 vs. CON and T2DM groups, respectively; ^++^ *p* < 0.01 vs. T2DM-SG group.

**Table 3:** The homeostasis model assessment of insulin resistance (HOMA-IR) index at week 12 (end of the experiment) in each group: CON (normal control), T2DM (type 2 diabetic rats), T2DM-SG (type 2 diabetic rats administered 10 mg/kg B.W. sitagliptin), and T2DM-MLE (type 2 diabetic rats administered 40 mg/kg B.W. mango leaf extract).

HOMA-IR	
CON	0.39 ± 0.01
T2DM	10.68 ± 0.75 ***
T2DM-SG	3.59 ± 0.12 ^###^
T2DM-MLE	3.71 ± 0.33 ^###^

The data are presented as mean ± SEM (n = 8 per group). ***, ^###^ *p* < 0.001 vs. CON and T2DM, respectively.

## Data Availability

The original contributions presented in this study are included in the article. Further inquiries can be directed to the corresponding authors.

## Appendix A

**Table A1:** Binding energy (kcal/mol) of 27 bioactive compounds against diabetes-associated target enzymes.

Compound	BE (kcal/mol)			Compound	BE (kcal/mol)		
	GSK-3β	DPP-IV	GLP-1R		GSK-3β	DPP-IV	GLP-1R
Reference inhibitor	−9.28	−10.94	−8.71	Catechin	−7.35	−6.71	−7.24
2,4′,6-trihydroxy-4- methoxy benzophenone	−6.33	−6.27	−6.28	Kaempferol 3-*O*-rutinoside	−8.13	−6.84	−5.35
3-glucosylmaclurin	−6.52	−6.61	−5.35	Hyperin	−7.99	−6.96	−5.16
Caffeic acid	−6.08	−4.88	−5.41	Isoswertisin	−9.30	−4.57	−4.91
Ellagic acid	−7.61	−6.45	−5.58	Isovitexin	−7.75	−8.15	−5.09
Gallic acid	−3.99	−3.39	−5.47	Quercetin	−7.06	−6.62	−5.28
Hypericin	−12.62	−7.45	−5.77	Rhamnetin	−7.57	−6.77	−5.00
Kainic acid	−4.45	−5.33	−4.15	Taxifolin	−7.02	−6.53	−5.20
Maclurin	−6.27	−6.22	−4.94	Lupeol	−9.93	−7.77	−7.29
Photocatechiuc acid	−4.17	−4.34	−5.53	Mangiferin	−6.76	−6.47	−3.83
Shikimic acid	−4.56	−3.89	−4.20	Mangiferonic acid	−11.45	−9.10	−9.08
Theogallin	−6.49	−6.77	−6.24	Norathyriol	−6.78	−5.49	−5.02
Amentoflavone	−10.15	−9.09	−6.68	Stigmasterol	−10.22	−9.05	−6.87
Apigenin	−7.13	−6.71	−6.21	Taraxeral	−9.96	−9.18	−8.27

**Table A2:** Drug- likeness properties prediction for 27 compounds.

Compound	Molecular Weight (g/mol) < 500	MLogP < 5	Hydrogen Bond Donor < 5	Hydrogen Bond Acceptor < 10	Rotatable Bond < 10
2,4′,6-trihydroxy-4- methoxy benzophenone	260.24	0.84	3	5	3
3-glucosylmaclurin	424.36	−2.77	9	11	4
Caffeic acid	180.16	0.70	3	4	2
Gallic acid	170.12	−0.16	4	5	1
Ellagic acid	302.19	0.41	4	8	0
Hypericin	504.44	1.36	6	8	0
Kainic acid	213.23	−1.98	3	5	4
Maclurin	262.21	0.04	5	6	2
Photocatechiuc acid	154.12	0.40	3	4	1
Shikimic acid	174.15	−1.43	4	5	1
Theogallin	344.27	−1.71	7	10	4
Amentoflavone	538.46	0.25	6	10	3
Apigenin	270.24	0.52	3	5	1
Catechin	290.27	0.24	5	6	1
Hyperin	464.38	−2.59	8	12	4
Isoswertisin	446.40	−1.80	6	10	4
Isovitexin	432.38	−2.02	7	10	3
Kaempferol 3-*O*-rutinoside	594.52	−3.43	9	15	6
Quercetin	302.24	−0.56	5	7	1
Rhamnetin	316.26	−0.31	4	7	2
Taxifolin	304.25	−0.64	5	7	1
Lupeol	426.72	6.92	1	1	1
Mangiferin	422.34	−2.66	8	11	2
Mangiferonic acid	454.68	5.73	1	3	5
Norathyriol	260.20	−0.24	4	6	0
Stigmasterol	412.69	6.62	1	1	5
Taraxeral	426.73	7.07	1	1	0
